# A qualitative study on stakeholders’ views on the participation of pregnant women in the APOSTEL VI study: a low-risk obstetrical RCT

**DOI:** 10.1186/s12884-019-2209-7

**Published:** 2019-02-11

**Authors:** Indira S. E. van der Zande, Rieke van der Graaf, Martijn A. Oudijk, Elsbeth H. van Vliet-Lachotzki, Johannes J. M. van Delden

**Affiliations:** 1 Department of Medical Humanities, Julius Center for Health Sciences and Primary Care, University Medical Center Utrecht, P.O. box 85500, 3508 GA Utrecht, the Netherlands; 2 Department of Obstetrics and Gynaecology, Academic Medical Center, Amsterdam, the Netherlands; 3 Dutch Genetic Alliance (VSOP), Soest, the Netherlands

**Keywords:** Inclusion in research, Obstetrics and Gynaecology, Pregnant women, Research ethics, Willingness, Recruitment

## Abstract

**Background:**

Bioethicists argue that inclusion of pregnant women in clinical research should be more routine to increase the evidence-base for pregnant women and foetuses. Yet, it is unknown whether pregnant women and others directly involved are willing to be routinely included. Therefore, we first need to establish what these stakeholders think about research participation in regular pregnancy-related research. However, studies on their views are scarce. In our study, we piggy-backed on a relatively conventional RCT, the APOSTEL VI study, to identify the views of stakeholders on inclusion of pregnant women in this study.

**Methods:**

We conducted a prospective qualitative study using 35 in-depth semi-structured interviews and one focus group. We interviewed pregnant women (*n* = 14) recruited for the APOSTEL VI study, in addition to healthcare professionals (*n* = 14), Research Ethics Committee members (RECs) (*n* = 5) and regulators (*n* = 7) involved in clinical research in pregnant women.

**Results:**

Three themes characterise stakeholders’ views on inclusion of pregnant women in the APOSTEL VI study. Additionally, one theme characterises stakeholders’ interest in inclusion of pregnant women in clinical research in general. First, pregnant women participate in the APOSTEL VI study for potential individual benefit and secondarily for altruistic motives, contrary to hypothetical studies. Second, a gatekeeping tendency hampers recruitment of pregnant women who might be eligible and willing, and questions about pregnant women’s decisional capacities surface. Third, healthcare professionals sometimes use the counselling conversation to steer pregnant women in a direction. Fourth, all stakeholders are hesitant about inclusion of pregnant women in clinical research in general due to a protective sentiment.

**Conclusions:**

Pregnant women are willing to participate in the APOSTEL VI study for potential individual benefit and altruistic motives. However, an underlying protective sentiment, resulting in gatekeeping and directive counselling, sometimes hampers recruitment in the APOSTEL VI study as well as in clinical research in general. While bioethicists claim that inclusion of pregnant women should be customary, our study indicates that healthcare professionals, regulators, RECs and pregnant women themselves are not necessarily interested in inclusion. Advancing the situation and increasing the evidence-base for pregnant women and foetuses may require additional measures such as investing in the recruitment and feasibility of RCTs and stimulating pregnant women’s decisional capacities.

**Electronic supplementary material:**

The online version of this article (10.1186/s12884-019-2209-7) contains supplementary material, which is available to authorized users.

## Background

For decades, bioethicists, pharmacologists, researchers, clinicians and regulators have argued that research participation of pregnant women is essential in order to increase the evidence-base for drugs and treatments for their population, which is needed to achieve fair healthcare opportunities and overcome current suboptimal care and under-treatment [1–10]. Various efforts to challenge pregnant women’s underrepresentation in clinical research have been undertaken by the research community. The Unites States Office of Research on Women’s Health (ORWH) of the Department of Health and Human Services (DHHS) has endorsed the view that pregnant women are to be presumed eligible for participation in clinical research [11]. Another example is the Second Wave Initiative which was launched in 2009, a collaborative academic initiative to find ethically and scientifically responsible means to increase the knowledge base for the treatment of pregnant women with medical illness [1]. Some ethical guidelines have also made an attempt to promote fair inclusion of pregnant women, for example by stating that research in pregnant women should be encouraged [6] or that women should not be inappropriately excluded from research solely because they are pregnant [12]. Others have even argued for the routine inclusion of pregnant women, referring to the regular inclusion of pregnant women in both obstetric and non-obstetric potentially beneficial clinical research (potentially beneficial for the group and/or the individual), except when there are compelling scientific or ethical reasons to exclude them [13, 14]. The term ‘routine inclusion’ is not hitherto defined, but one proposal is to instigate stand-alone Phase I trials that begin at the same time as Phase III trials in the general population, or to instigate Phase I trials embedded into late Phase II or Phase III trials in the general population [13].

Nevertheless, research participation of pregnant women remains a complex issue, since inclusion of the woman also means inclusion of the foetus, with possibly far-reaching consequences for the future child. So while some bioethicists and guidelines claim that exclusion of pregnant women should be justified or that these women should be routinely included, for ethical reflection on practice it is essential to also identify the considered moral judgements of stakeholders who are directly involved in clinical research in pregnant women. However, literature on stakeholders’ views is presently scarce and there is uncertainty as to whether pregnant women themselves would be interested in participation in clinical research, even if they were found to be eligible. In the current literature on pregnant women’s willingness to participate in research, it appeared that they report similar motivations as non-pregnant research subjects in their reasoning about participation. For instance, reported reasons for participation were altruistic and personal motives whereas reported reasons for refusal primarily focused around inconveniences [15–20]. However, the majority of these studies employed either retrospective or hypothetical methods, which are problematic due to their recall bias and the gap between reported and actual behaviour.

In addition to continuing ambiguity regarding pregnant women’s willingness to participate, there are also concerns whether other stakeholders such as research ethics committee members (RECs) and physician-researchers are willing to include them. Currently, the literature indicates that even though RECs and researchers recognise the value of clinical research in pregnant women, they might be hesitant to conduct or promote research in pregnant women [20–25]. One underlying reason may be that recruitment and retention of pregnant women poses specific challenges, with choices around childbirth potentially being highly emotionally charged for both women and clinicians [24]. Apart from recruitment issues, there may also be additional reasons for stakeholders’ reluctance. The aim of our paper was to explore what stakeholders think about inclusion of pregnant women in the APOSTEL VI study: a low-risk obstetrical randomised controlled trial (RCT). In an effort to understand pregnant women’s reasoning while being confronted with an actual recruitment scenario, we conducted our in-depth interviews with pregnant women shortly after they had decided upon enrolment, thereby avoiding the drawbacks common to retrospective and hypothetical studies. Moreover, we explicitly piggy-backed on a relatively conventional obstetrical study, where risks were low and where potential individual benefit was not by itself a reason why every pregnant woman would automatically participate. As such, the decision to participate was not an obvious one and the respondents had to critically consider their choice with regard to participation.

## Methods

### Study design

We employed a qualitative study design using semi-structured in-depth interviews and one focus group to explore stakeholders’ views on the topic of inclusion of pregnant women in the APOSTEL VI study (trial registration number: NTR4210). Because our aim was to explore the context and the attitude and beliefs of stakeholders beyond the medical outcomes of the APOSTEL VI study, we chose in-depth interviews as the primary method of investigation. We conducted an exploratory focus group before instigating the interviews, in order to explore the topic among professionals and restructured the interview questions where necessary. The qualitative study presented here was part of a larger study, the Methods of which are described elsewhere [26]. We used the same research population for both studies, however, we aimed to answer two different research questions. This study focuses on the willingness and underlying motivations to include pregnant women in clinical research.

### Sample and setting

Throughout the larger study, we sought to reach maximum variation in context and conducted the study among a variety of stakeholders whom were contacted by the researcher. We explored the topics through interviews with four groups: pregnant women, healthcare professionals, REC members and regulators. We recruited pregnant women (*n* = 14) from the University Medical Center Utrecht (UMC Utrecht) and the Academic Medical Center (AMC) in Amsterdam, the Netherlands. Pregnant women were eligible when they were recruited for the APOSTEL VI study and had made their decision about enrolment in that study (see Additional file 1: Box S1.). As such, we included women who decided to participate in the study as well as women who declined participation in the study. At the time, the APOSTEL VI study was the only obstetrical study in the Netherlands that provided us access to the purposive sample of pregnant women recruited for a clinical study and the possibility to prospectively interview them with regard to the overarching research question of the larger study (*what are the views of stakeholders regarding pregnant women’s participation in clinical research?*) as well as the specific theme that is presented in this study, namely the *willingness* to include pregnant women. Accordingly, shortly after the women had decided about enrolment in the primary study, they were approached by research midwifes at the study sites. When they indicated an interest in our qualitative study they were later contacted by the researcher of the qualitative study and asked to participate in an interview. We interviewed the respondents after they were randomised to either perceive the pessary or no intervention.

**Fig. 1:**
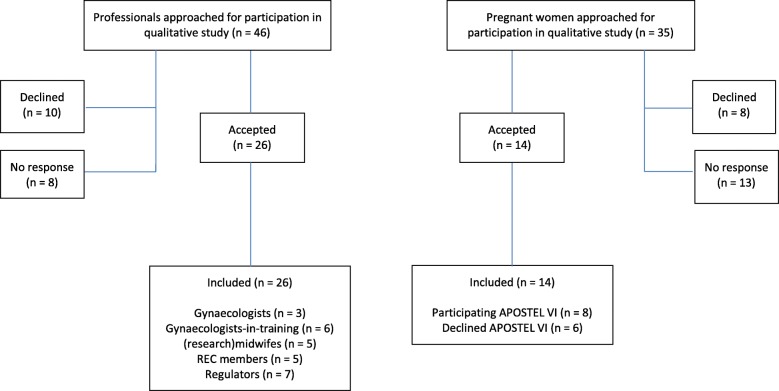
Flowchart of Inclusion

As described elsewhere, healthcare professionals and REC members were recruited from the two previously mentioned academic hospitals in the Netherlands [26]. We interviewed gynaecologists (*n* = 3), gynaecologists-in-training (*n* = 6), (research) midwifes (*n* = 5), and REC members (*n* = 5). Of the five REC members, two were also gynaecologists. Additionally, we organised one focus group of 1:15 h with regulators (*n* = 5) from LAREB, a Dutch pharmacovigilance centre, where we spoke with employees from the Teratogenic Information Service (TIS) department. Finally, we interviewed two regulators from the Dutch Medicine Evaluation Board (MEB). The ethical framework that guides professionals in the Netherlands primarily consists of the national legislation (Medical Research Involving Human Subject Act (WMO)) and additionally of international guidance documents such as the Declaration of Helsinki. The WMO has offers no specific guidance on research involving pregnant women. See Tables 1 and 2 with characteristics of participants and Fig. 1 with the flowchart of inclusion. The REC of the UMC Utrecht assessed the qualitative research proposal and issued a waiver for the project.

**Table 1:** Demographic characteristics pregnant women

Characteristics pregnant women	(*n* = 14)
Age	
< 25	1
25–30	5
31–40	8
Parity	
Nulliparous	9
Primiparous	2
Multiparous	3
Gestational age (weeks)	
25–30	5
31–35	9
Education	
Highschool	3
Lower vocational (MBO)	3
College (HBO/WO)	4
Graduate degree	4
Partner	
Married	5
Living together	9
Ethnicity	
Dutch	11
Dutch/Indonesian	1
Surinamese	1
Polish	1
Enrollment in study	
Participating in Apostel VI - *Recruited from UMC Utrecht* - *Recruited from AMC*	8 *3* *5*
Not participating in Apostel VI - *Recruited from UMC Utrecht* - *Recruited from AMC*	6 *6* *0*

**Table 2:** Demographic characteristics professionals

Characteristics professionals	(*n* = 26)^a^
Gender	
Male	11
Female	15
Age	
25–40	13
41–55	7
> 55	6
Experience at present job (years)	
< 5	13
5–10	6
11–15	4
16–20	3
Profession	
Gynaecologist	3
Gynaecologist-in-training^b^	6
Midwife^c^	5
REC member^d^	5
Regulator/knowledge centre	7

^a^5 regulators from the focus group, 21 interviewees

^b^1 gynaecologist-in-training was a gynaecologist-not-in-training (ANIOS)

^c^3 clinical research midwifes based in academic hospitals

^d^2 REC members were also gynaecologists

### Data collection and analysis

As the data collection and analysis was carried out for the larger study as a whole, the process was similar as the one described elsewhere [26]. In short: all participants were interviewed by one researcher (IvdZ). The focus group was conducted by two researchers (IvdZ and RvdG). Verbal informed consent and written informed consent in case of the pregnant women was obtained from all participants. Initial interview topics and questions were formulated after examination of the relevant literature and discussion with members of the team (see Table 3 for the general topic list and Additional file 2 and Additional file 3 for examples of extended topic lists). The semi-structured in-depth interviews were conducted according to a predefined topic list, however, according to the technique of constant comparative analysis, the interview topics evolved as the interviews progressed through an iterative process where the desired result is reached by repeating rounds of analysis [27]. Interviews took place at the workplace or the home of the respondents. Thematic saturation was reached after 20 interviews. Data collection took place from March 2015 to September 2016.

**Table 3:** General Topic List

• Participation of pregnant women in clinical research; • Routine inclusion in clinical research; • Balancing risks and potential benefits; • Conflict of interest maternal-foetal benefit; • Whose interests should prevail; • Societal benefit versus therapeutic benefit; • Recruitment process at the medical centre; • Balancing risks and potential benefits in the APOSTEL VI; • Decision-making process in the APOSTEL VI.	

The data analysis was carried out according to the thematic analysis method [28, 29]. The focus group and the interviews were transcribed verbatim and the data was imported in the software programme Nvivo 10 [30]. IvdZ independently coded the transcripts and through comparison across transcripts higher order themes were found. RvdG checked codes for consistency and the found themes were discussed at team meetings until a consensus was reached. To enhance the validity of our findings, we organised an expert meeting in the last phase of data collection in which we discussed whether our results were an accurate representation of the practice of clinical research in pregnant women. During the expert meeting, we discussed the larger study as a whole, as well as the findings relating to stakeholder’s views on the inclusion of pregnant women in clinical research, as presented here.

## Results

Based on the responses of the interviewees, we were able to identify three themes characterising stakeholder’s views on the inclusion of pregnant women in the APOSTEL VI study. Additionally, we identified one theme that characterised stakeholders’ views on the inclusion of pregnant women in clinical research in general. These themes emerged consistently in one way or another in all interviews. Per theme, the views of pregnant women, healthcare professionals, REC members and regulators are presented. The starting point of the interviews was the APOSTEL VI study, but respondents sometimes extended their observations to include research participation in general, in which cases we included that in the theme as well. Moreover, the fourth theme as a whole relates to participation of pregnant women in clinical research in general. Representative quotations were chosen in order to illustrate the identified themes (Table 4).

**Table 4:** Representative quotations

Theme	Quotations^a^
Motivations for participation	PW02, participating in APOSTEL VI: *I like to participate when it is positive for me, when participation makes me feel like I do something good, but that it is also positive for myself and that nothing can go wrong.* PW12, not participating in APOSTEL VI: *It depends whether participation is beneficial for yourself or whether it is purely for science. In this case, I considered it a valuable bonus that the pessary could potentially help to prolong my pregnancy.* PW13, not participating in APOSTEL VI: *[Healthcare professionals] did not want to perform internal examinations to prevent stimulation of the uterus. So I figured, if you insert a pessary, then you can also stimulate it. I was afraid of that.* HCP02, research midwife: *It is first and foremost herself and the baby. Saving the world comes secondary.* HCP04, gynaecologist-in-training: *Why would you do an intervention, why would we do something that has not been proven? I also wonder what the working mechanism of the pessary is, nobody can tell me, not even the big advocates.*
Counselling	HCP13, gynaecologist-in-training: *It is an easy study to recruit people for, because it involves people who really want something, and you have something to offer.* HCP05, gynaecologist: *You can only achieve fair inclusion when you ask each and every pregnant woman who potentially meets the inclusion criteria.* HCP10, research midwife: *If they are eligible, we ask them. In the following conversation I may determine that they are not suitable, for example because they do not understand it [the study]. We also have drug addicts here, where you decide that it is not a good idea because that lady doesn’t belong to the group of women the study is interested in.* HCP08, midwife: *If there is a study where you think ‘I’m not sure what I’m doing here’, it is definitely a reason to counsel in the other direction. You try to counsel objectively, but we all know it is directive.* HCP13, gynaecologist-in-training: *Sometimes you know that it is not the right candidate. That it will be a mess. And then you counsel slightly more negatively.*
Gatekeeping	PW07, participating in APOSTEL VI: *I had so many doubts, I really didn’t know. You are as mentally unstable as it can be when you lay there hospitalised so I couldn’t make a good decision. Yes, many people where involved [in the decision-process].* PW11, participating in APOSTEL VI: *I notice that you think different about things when you are pregnant, it may be hormonal or not, but you are surely different in terms of decisiveness in comparison to when you’re not pregnant.* PW03, not participating in APOSTEL VI: *Sometimes I realise that I am less resolute in my decisions because I am pregnant. […] More doubtful and no completely following, like “oh no, what was it [that I missed]?”. For that reason I turn to other people.* PW08, not participating in APOSTEL VI: *Everyone makes you aware of the fact that, as a pregnant woman, you are part of a weaker group. That you should be handled with great care.* HCP01, research midwife: *A dad finds it burdensome: ‘there they are yet again with a study; she is already tired, she is sleeping, no, I don’t think that she will participate’.* REC05, gynaecologist: *I notice that clinicians are protective towards patients, for example in that they do not mention ongoing scientific research.* HCP08, midwife: *Sometimes we ourselves decide that someone is not suitable. Because of the language, or when you wonder whether someone will understand it, or because someone is already participating in two other studies.* HCP03, gynaecologist-in-training: *The child cannot decide if he wants to participate in a potentially dangerous study. […and a pregnant woman] cannot estimate or oversee the risks for a child that may has to become 80 years old.* HCP14, gynaecologist: *Assuming that women may function differently during their pregnancy, also psychologically, you don’t know if that does not influence their decision-making surrounding research participation.* HCP13, gynaecologist-in-training: *I think they [pregnant women] are behaviourally more vulnerable. I think they have some sort of black, blind spot: everything for the child. […] They are not sufficiently competent.*
Interest in (routine) inclusion	HCP05, gynaecologist: *Routine inclusion may be a little odd, but if you have the premise that there is a theoretical or practical basis to assume that a given therapy improves or can improve the pregnancy outcome, and you meet the strict guidelines of among others the REC and the WMO [Dutch regulation on the protection of human subjects], and you carefully register the outcome of the pregnancy and the side effects, I think that that would actually be very good.* HCP03, gynaecologist-in-training: *The question is whether there are no good alternatives. Is research really necessary?* REG01, MEB member: *Observational research has a different approach, where we do not intentionally expose pregnant women, but where women are already exposed and we try to collect data in the best way.* REC04, clinician: *You should not expose pregnant women to medications of which the effects on the baby are unknown, if you have an alternative. It’s different if it is pregnancy-specific. In that case you don’t have an alternative, and then I have fewer objections.* PW02, participating in APOSTEL VI: *I would not participate in a study where I have to take medications or where things are injected into me. I don’t want to be a guinea pig for that.* PW12, not participating in APOSTEL VI: *If possible, I would not accept any research with risks. Why would you take risk if you don’t have to, if there is no direct benefit? I wouldn’t take that risk for science.*

^a^ Quotations are sometimes slightly modified in order to enhance readability

### Theme 1. Motivations for participation

The interviews with pregnant women demonstrated that they experienced the decision process about enrolment in the APOSTEL VI study as difficult, also in light of their particular situation: a healthy pregnancy up till the moment they were suddenly admitted to the hospital for threatened preterm birth and having to decide upon study enrolment. Six women were pregnant for the first time, while of the other eight women, seven had had one or more miscarriages in the past. The medical history did not seem to have an effect on participation. Pregnant women who chose to participate reasoned that participation could potentially be beneficial for the foetus (preventing a preterm birth) while they perceived there to be no risk since the pessary would not reach the foetus in any way. Another mentioned reason for participation was an altruistic motive to help future women and children and to advance science in general, although this reason was secondary and most women mentioned that they would not have participated if there was no potential benefit.

Reported reasons for refusal were the required extra internal exam that was necessary before the eligibility for participation in the RCT was established (only in UMC Utrecht) which was perceived to be risky, and the extra anxiety or stress that actual participation would entail, stemming from a fear of the pessary itself or the possible consequences of the device (e.g. excessive secretion, or stress of having the pessary). With regard to their risk-benefit assessment, most pregnant women indicated that they perceived the APOSTEL VI study to pose zero risk (*n* = 12) because enrolment would not negatively impact the development or growth of their child whereas they found the burdens relatively small [26].

Healthcare professionals confirmed the reasons that pregnant women mentioned about their enrolment decision, thereby emphasising that they perceive pregnant women’s altruistic reasons to be secondary to motivations of potential individual benefit. Additionally, healthcare professionals voiced concerns about the risks of the APOSTEL VI study and additional concerns with regard to the actual working mechanism of the pessary, the pessary itself and the extra internal exam [26]. Despite these concerns, most healthcare professionals mentioned that as the APOSTEL VI study was not perceived to be harmful, they were generally positive about inclusion of pregnant women in the APOSTEL VI study.

### Theme 2. Gatekeeping

In light of pregnant women’s reported uncertainty with regard to participation in the APOSTEL VI study, most pregnant women (*n* = 12) mentioned that they consulted with others about their enrolment decision and said that third party advise was important to them. A decision was usually made in deliberation with at least the partner, and sometimes included additional conversations with other family members or healthcare professionals. Pregnant women mentioned that in these conversations with others, they perceived their partners as well as society in general to be particularly protective towards them and their future child. In interviews with other stakeholders, it appeared that the main reason why stakeholders are reluctant to include pregnant women in clinical research stems from this felt need to protect the woman and her foetus and not overburden her during her pregnancy. In other contexts, this phenomenon has been called gatekeeping. To illustrate, healthcare professionals reported that they sometimes decide not to ask pregnant women to participate in clinical research because they perceive a study not to be in their patient’s interest; they believe the number of studies their patient is participating in is already sufficient and do not want to overload them; or they do not want to overburden their patients in relation to their personal situation (such as living circumstances or being a single parent) or simply overburden them in general. REC members and regulators furthermore mentioned the responsibility they felt for protection of pregnant women and their foetuses.

When asked about underlying reasons for the protective attitude towards pregnant women, a noticeable element that a number of respondents kept referring to was pregnant women’s decision-making capacity, especially with regard to risk assessment. As mentioned above, doubts about their decision-making capacities were also mentioned by pregnant women themselves (“feeling too unstable to make the right decision”), who, possibly in light of their own doubts about being able to make the right decision, mentioned that they more often looked for third party advice. There appeared to be a general feeling among interviewees that there might be moments when the decision-making capacity of pregnant women might be threatened due to for example “being very emotional” or “completely stuffed with hormones”, and that they might need protection for that reason. Another reason that was mentioned, was the societal idea of “being a perfect mother” and “having a blind spot for the baby” which respondents felt could influence the behaviour of pregnant women, both in relation to daily life as well as to participation in clinical research.

### Theme 3. Counselling

The interviews with healthcare professionals showed that the recruitment process in obstetric research is focused around what they call the “counselling” conversation. As such, when a pregnant woman is found to be eligible for participation in the APOSTEL VI study, the first step is to initiate a counselling conversation in which she is informed about the study. The counselling conversation appeared to be decisive for pregnant women who said that they based their understanding of the APOSTEL VI study primarily on the conversation rather than patient information forms. Healthcare professionals confirmed the importance of the counselling conversation and specified that they perceived the counselling for the APOSTEL VI study to be simple because a) the potential participants really want something and you have something to offer, b) APOSTEL VI is one of the easiest studies to explicate, c) it is a relatively innocent study because you can always remove the pessary without any harm and d) the logistics are easy since the women are already hospitalised and there is time for a conversation. Counselling for the APOSTEL VI study was thus found to be straightforward and feasible.

Healthcare professionals, in their role as researchers, reported a clear notion of the concept of counselling: the conversation should be objective and all eligible pregnant women should be asked to participate. When asked about counselling of their own patients, some healthcare professionals seemed to deviate from the described notion of counselling. To illustrate, counselling sometimes appeared to be used as a way to direct patients in a certain way regarding the decision whether or not to enrol in clinical research. For example, at times it may happen that healthcare professionals are hesitant to include their patients and make a conscious choice to “counsel negatively”. As was mentioned in the previous theme, most reasons for negative counselling concerned a fear of overburdening their patients. However, another reason that was mentioned was the research interest itself, when the professionals for example believe that their patient is not the right candidate for the study or they believe that the study itself is not relevant. Counselling also appears in opposite direction, when the conversation is used to motivate patients to participate, referred to as “counselling positively”, for example by negating or downplaying possible negative effects of research participation.

### Theme 4. Interest in (routine) inclusion

While the starting point of the interviews was the APOSTL VI study, respondents additionally articulated their views on research participation of pregnant women in clinical research in general. As such, the interviews demonstrated that all stakeholders are cautious when asked about inclusion of pregnant women in clinical research, and especially hesitant about routine inclusion. Since the term “routine” is undefined, we provided respondents with different examples where pregnant women could be included, thereby making a difference between obstetric and non-obstetric; interventional (e.g. experimental research or research about standard care practices) and observational research. We always specified that “routine” was not a universally agreed upon or currently practised term, but that we meant a general reference to having a default of inclusion of pregnant women unless there are scientific and ethical reasons for their exclusion [13].

Healthcare professionals reported an interest in inclusion of pregnant women in RCTs, as long as research participation would potentially be beneficial for the group or the individual or at least have no or no negative effect on the individual. REC members and regulators reported a preference for inclusion of pregnant women in observational research and mentioned that inclusion in RCTs should only be an option when it comprises research with demonstrated necessity (“there are really no alternatives”) and a real potential for improvement of the pregnancy outcome of the individual woman. To illustrate, they mentioned that if there is a new medication that might be better than known safe alternatives (including long time used off-label medications), they would not want a trial with the new medication because in a trial there would be a risk of taking a medication of which the safety is not entirely established, but rather continue with the (off-label) alternatives. Contrarily, if a medical treatment would be indicated and the effectiveness of different standard care practices needed to be proven, respondents found inclusion more acceptable because the risks would be known. Moreover, the respondents made a clear distinction between obstetric and non-obstetric research, arguing that inclusion in obstetric research was more “defendable” and added that women (in the Netherlands) who are ill are often already included in obstetric research performed by the Dutch Consortium for Healthcare Evaluation and Research in Obstetrics and Gynecology, NVOG consortium. With regard to further inclusion of pregnant women in clinical research, respondents mentioned that there was room for inclusion in specific cases, but that they had no desire to routinely include pregnant women in the way we had defined the term.

Most pregnant women (*n* = 11) mentioned that they would only consider research participation during their pregnancy in case such research was imperative for their health condition. As an example, they mentioned having a certain illness for which research participation would offer high potential personal benefit. Especially with regard to invasive clinical research (which they characterised as being experimental; concerning medications or injections of some sort; or entailing the internal insertion of a medical device) there was no willingness to participate. With regard to non-invasive observational research, pregnant women mentioned that they would be willing to participate to help future pregnant women, as long as inconveniences such as time requirements were not too extensive. Additionally, pregnant women reported an interest in the development of registries in which pregnant women would automatically participate in order to increase the evidence-base, but they were reluctant with regard to routine inclusion in trials. To clarify, they mentioned that research on medical conditions for which there are really no treatment options and the risks of the condition for the woman and the foetus are very high would be understandable, but research on new medications for medical conditions for which there are safe alternatives (including off-label medications) would not be preferable.

## Discussion

Our qualitative study shows that there are different reasons why pregnant women in the Netherlands are willing to participate in the APOSTEL VI study, a relatively conventional low-risk obstetrical RCT. In contrast to earlier hypothetical studies where altruism was identified as the primary motivator, our study indicates that women who are confronted with an actual recruitment scenario are primarily motivated by potential individual benefit, while altruistic motives are secondary. Because of uncertainty about pregnant women’s reasons for participation, there are assumptions that pregnant women may have different reasons for participation than non-pregnant participants [3, 20, 31]. Nevertheless, pregnant women in our study actually report similar reasons for participation as non-pregnant participants, where altruistic motivations were also said to be secondary to potential individual benefit [32, 33]. With regard to altruism, it is particularly interesting that in our case, where pregnant women are fairly desperate and unsurprisingly mention potential individual benefit as a primary reason for participation, altruism is still cited as a secondary reason. Similar to reports from earlier low-risk studies, one of the main reasons to refuse participation in the APOSTEL VI was physical inconvenience, in this case the required extra internal exam [34, 35]. In that respect, the APOSTEL VI exemplifies the importance of managing practicalities. To illustrate, there was a clear difference in recruitment numbers between the two academic centres: UMC Utrecht: 18/40 persons included/not included versus AMC 49/15 included/not included. At the UMC Utrecht, healthcare professionals had voiced concerns about the APOSTEL VI study beforehand and may have (unconsciously) conveyed these concerns to potential research subjects. Moreover, the internal exam was, contrarily to the practice at the AMC, not part of standard care and thus became an extra invasive procedure which was considered to be a barrier for both pregnant women and healthcare professionals.

Furthermore, while bioethicists claim that inclusion of pregnant women in clinical research should be promoted or should even be routine, our qualitative study shows that stakeholders in the Netherlands are not necessarily interested in (routine) inclusion of pregnant women in clinical research, unless there are specific pressing cases in which the potential individual benefits are very high. Illustrative is the hesitance to conduct clinical research on new medications and the preference for continuing with long-time used off-label medications, which are arguably not well-researched or have long-term follow-up data. The underlying reason for the reluctance to include pregnant women seems to be a protective sentiment, which possibly explains the continuous underrepresentation and constant recruitment and delay struggles. The protective sentiment relates to the theme of gatekeeping. First, on an individual level, healthcare professionals, RECs, regulators and possibly partners of pregnant women and pregnant women themselves appear to be resolved to shield women from any form of harm. Alarmingly, questions about pregnant women’s competence to make decisions about research participation keep surfacing, questions that were also raised by pregnant women themselves. While finding a decision hard to make or consulting others for help are features of difficult decisions rather than an indication of issues with decision-making capacity, the particular questions related to pregnant women’s ability to make decisions throughout the pregnancy, noticeably exceeding the matter of merely finding a decision hard to make.

Second, on a professional level, our study confirms earlier findings that a sometimes paternalistic tendency for protection, demonstrated in the exclusion of possibly eligible pregnant women for their own good, hampers recruitment [10, 21, 36, 37]. Gatekeeping by healthcare professionals could be facilitated by the current nature of the counselling process, which results in two problems. First, when healthcare professionals make an individual selection of patients instead of including every eligible patient, a bias is introduced in the research population, possibly infringing upon the scientific validity of clinical research. Second, patients’ informed choice is threatened when clinicians steer patients in a direction they deem suitable.

Evidently, healthcare professionals should be entrusted to prevent individual patients from participating if they deem participation harmful. Nevertheless, the practice of gatekeeping on a structural level extends far beyond protection of an individual patient because it results in the exclusion of groups of possibly eligible pregnant women, which is problematic. Striking a balance between respecting pregnant women’s competence to make an informed decision and not overly protecting them on the one hand, and safeguarding them from harm on the other is essential. Yet, protection can also mean *inclusion* in clinical research, thereby gathering evidence that may place fewer pregnant women and their foetuses at risk than the much larger number of pregnant women who will be exposed to the medications once they come to market [9]. The current lack of specific guidance for research with pregnant women, for example in the WMO, may contribute to uncertainty about the preferred course of action with regard to inclusion of pregnant women. Providing specific guidance is therefore commendable.

Our qualitative study shows that research participation of pregnant women remains a charged topic and that it is unlikely that routine inclusion will become the norm overnight. Even with regard to our low-risk obstetric study, stakeholders were hesitant about inclusion of pregnant women. Imaginably, this hesitance is increased when it concerns non-obstetric research, the area where research is most problematically lacking interventions and medications that treat or prevent maternal and foetal illness during pregnancy. Following from these results, we may want to invest in additional ways to increase the evidence base for pregnant women. For example, solutions could be found in sharpening or adjusting the recruitment process, based on pregnant women’s reasons for participation which, as the APOSTEL VI study exemplifies, are personal benefit and secondarily altruistic motives. Since reasons for participation are similar to those of non-pregnant research participants, lessons can be learned from recruitment strategies that have been used in these groups. Moreover, raising stakeholders’ awareness on their protective attitude and the resulting negative effects may contribute to the promotion of pregnant women’s decisional capacities. Another practical solution may also be to capitalise on feasibility, for example by asking healthcare professionals beforehand to assess the logistics and potential risks and benefits of a study, thereby decreasing the risk of delay and lack of equipoise which is often mentioned as a barrier for inclusion and which may have been the case in the APOSTEL VI [24, 31, 38].

### Limitations

This qualitative study has a number of limitations. First, we interviewed mostly highly educated stakeholders regarding only the Dutch situation and it is possible that the results are different in other countries, thus challenging the generalizability of the findings. Second, the saturation number of 20 interviews was reached on group level, but not always on sub-group level. As such, our inter-group comparisons are less valid than our group analyses. Third, we only included pregnant participants who were recruited for the APOSTEL VI study, a group that consists of women that become sick during their pregnancy and whom are recruited for a low-risk obstetric study. Future research should also aim to include research subjects from the group of sick women who become -or prepare to become- pregnant and participants recruited for high risk and non-obstetrical studies. We attempted to include women from the latter group, but all three trials we collaborated with were unfortunately cancelled, possibly another illustration of the gatekeeping tendency surrounding clinical research in pregnant women. Finally, we were unable to interview any representatives from a pharmaceutical company, since the seven organisations we contacted with a request to participate unfortunately did not respond or did not want to participate in our study.

## Conclusions

Our qualitative study shows that pregnant women are willing to participate in the relatively conventional low-risk obstetrical APOSTEL VI study for potential individual benefit and altruistic motives. But while pregnant women might be eligible and willing to participate, a protective sentiment seems to dominate the practice of the APOSTEL VI as well as clinical research in general. While bioethicists claim that inclusion of pregnant women in clinical research should be promoted or should even be routine, our study indicates that healthcare professionals, regulators, REC members and pregnant women themselves are not necessarily interested in inclusion unless there is a high potential for individual benefit. The underlying reason for the reluctance to include pregnant women appears to be a protective sentiment. This sentiment results in gatekeeping and directive counselling, threatening pregnant women’s informed choice and hampering recruitment of eligible and potentially willing participants. Striking a balance between respecting pregnant women’s autonomy and protecting them is essential.

## Additional files

Additional file 1: Box S1 a description of the APOSTEL VI study. (DOCX 22 kb)

Additional file 2: Table S1-A: Topic list healthcare professionals. (DOCX 23 kb)

Additional file 3: Table S1-B: Topic list pregnant women. (DOCX 24 kb)

## Abbreviations

AMC: Academic Medical Center Amsterdam
APOSTEL VI trial: Assessment of Perinatal Outcome after Specific Treatment in Early Labour
DHHS: Department of Health and Human Services
DSMB: Data and Safety Monitoring Board
HCP: healthcare professional
LAREB: Netherlands Pharmacovigilance Centre
MEB: Medicines Evaluation Board
NVOG: Dutch Consortium for Healthcare Evaluation and Research in Obstetrics and Gynecology
ORWH: Office of Research on Women’s Health
PW: pregnant woman
RCT: randomised controlled trial
REC: Research Ethics Committee (member)
REG: regulator
TIS: Teratology Information Service
UMC Utrecht: University Medical Center Utrecht

## References

[1] Lyerly AD, Little MO, Faden R (2008). The second wave: toward responsible inclusion of pregnant women in research. Int J Fem Approaches Bioeth.

[2] Little M, Lyerly A, Faden R (2009). Pregnant women and medical research: a moral imperative. Bioethica Forum.

[3] Baylis F (2010). Pregnant women deserve better. Nature.

[4] Haas DM, Gallauresi B, Shields K, Zeitlin D, Clark SM, Hebert MF (2011). Pharmacotherapy and pregnancy: highlights from the third international conference for individualized pharmacotherapy in pregnancy. Clin Transl Sci.

[5] EMA (European Medicines Agency) (2005). Guideline of the exposure to medicinal products during pregnancy: need for post-authorisation data.

[6] CIOMS & WHO (2015). CIOMS draft guidelines.

[7] Shields KE, Lyerly AD (2013). Exclusion of pregnant women from industry-sponsored clinical trials. Obstet Gynecol.

[8] Zajicek A, Giacoia GP (2007). Obstetric clinical pharmacology: coming of age. Clin Pharmacol Ther.

[9] Macklin R (2010). Enrolling pregnant women in biomedical research. Lancet.

[10] Frew PM, Saint-Victor DS, Isaacs MB, Kim S, Swamy GK, Sheffield JS (2014). Recruitment and retention of pregnant women into clinical research trials: an overview of challenges, facilitators, and best practices. Clin Infect Dis.

[11] Levine RJ (2011). IRB perspective on inclusion of pregnant women in clinical research. In: ORWH workshop: Enrolling pregnant women: Issues in clinical research. An ORWH 20th Anniversary Event.

[12] Secretariat on Responsible Conduct of Research. Tri-Council Policy Statement (TCPS-2): Ethical conduct for research involving humans. 2014. http://www.pre.ethics.gc.ca/eng/policy-politique/initiatives/tcps2-eptc2/Default/.

[13] Baylis F, Halperin SA (2012). Research involving pregnant women: trials and tribulations. Clin Investig (Lond).

[14] Noah BA (2014). The inclusion of pregnant women in clinical research barbara a. noah* a.

[15] Lyerly AD, Namey EE, Gray B, Swamy G, Faden RR (2012). Women’s views about participating in research while pregnant. IRB Ethics Hum Res.

[16] Vecchi Brumatti L, Montico M, Russian S, Tognin V, Bin M, Barbone F (2013). Analysis of motivations that lead women to participate (or not) in a newborn cohort study. BMC Pediatr.

[17] M a R, Makropoulos D, Walker M, Keely E, Karovitch A, Wells PS (2003). Participation of pregnant women in clinical trials: will they participate and why?. Am J Perinatol.

[18] Lavender T, Kingdon C (2009). Primigravid women’s views of being approached to participate in a hypothetical term cephalic trial of planned vaginal birth versus planned cesarean birth. Birth.

[19] Oude Rengerink K, Logtenberg S, Hooft L, Bossuyt PM, Mol BW (2015). Pregnant womens’ concerns when invited to a randomized trial: a qualitative case control study. BMC Pregnancy Childbirth.

[20] van der Zande ISE, van der Graaf R, Hooft L, van Delden JJM. Facilitators and barriers to pregnant women's participation in research: A systematic review. Women Birth. 2018;31(5):350-61. 10.1016/j.wombi.2017.12.009. 10.1016/j.wombi.2017.12.009 29373261

[21] Tooher RL, Middleton PF, Pregnancy CCABMC (2008). Childbirth a thematic analysis of factors influencing recruitment to maternal and perinatal. Trials.

[22] Blehar MC, Spong C, Grady C, Goldkind SF, Sahin L, Clayton JA (2013). Enrolling pregnant women: issues in clinical research. Women’s Heal Issues.

[23] Brandon AR, Shivakumar G, Inrig SJ, Sadler JZ, Craddock Lee SJ (2014). Ethical challenges in designing, conducting, and reporting research to improve the mental health of pregnant women: the voices of investigators and IRB members. AJOB Empir Bioeth.

[24] Madan A, Tracy S, Reid R, Henry A (2014). Recruitment difficulties in obstetric trials: a case study and review. Aust N Z J Obstet Gynaecol.

[25] Haas DM, Wunder K, Wolf JG, Denne SC (2010). Women’s health care providers’ attitudes toward research in pregnancy. J Reprod Med.

[26] Van Der Zande ISE, Van Der Graaf R, Oudijk MA, Van Delden JJM. A qualitative study on acceptable levels of risk for pregnant women in clinical research. BMC Med Ethics. 2017;18:35. 10.1186/s12910-017-0194-9. 10.1186/s12910-017-0194-9 PMC5432995 28506267

[27] Charmaz K. Constructing Grounded Theory: A Practical Guide through Qualitative Analysis. Constructing Grounded Theory: A Practical Guide Through Qualitative Analysis. London: SAGE Publications Ltd; 2006.

[28] Braun V, Clarke V (2006). Using thematic analysis in psychology. Qual Res Psychol.

[29] Thomas J, Harden A (2008). Methods for the thematic synthesis of qualitative research in systematic reviews. BMC Med Res Methodol.

[30] QSR. NVivo 10 research software for analysis and insight. 2014. www.qsrinternational.com.

[31] Mohanna K, Tunna K (1999). Withholding consent to participate in clinical trials: decisions of pregnant women. Br J Obstet Gynaecol.

[32] Edwards SJL, Lilford RJ. The ethics of randomised controlled trials from the perspectives of patients, the public, and healthcare professionals. BMJ. 1998;317(7167):1209–212. 10.1136/bmj.317.7167.1209 PMC1114158 9794861

[33] McCann SK, Campbell MK, V a E (2010). Reasons for participating in randomised controlled trials: conditional altruism and considerations for self. Trials.

[34] van Delft K, Schwertner-Tiepelmann N, Thakar R, Sultan a H (2013). Recruitment of pregnant women in research. J Obstet Gynaecol.

[35] Mihrshahi S, Vukasin N, Forbes S, Wainwright C, Krause W, Ampon R (2002). Are you busy for the next 5 years? Recruitment in the childhood asthma prevention study (CAPS). Respirology.

[36] Helmreich RJ, Hundley V, Norman A, Ighedosa J, Chow E (2007). Research in pregnant women: the challenges of informed consent. Nurs Womens Health.

[37] Mccauley-elsom K, Gurvich C, Lee S, Elsom S, Connor MO, Kulkarni J (2009). Vulnerable populations and multicentred research.

[38] Turner CE, Young JM, Solomon MJ, Ludlow J, Benness C, Phipps H (2008). Willingness of pregnant women and clinicians to participate in a hypothetical randomised controlled trial comparing vaginal delivery and elective caesarean section. Aust New Zeal J Obstet Gynaecol.

