# Supplementary material for: A qualitative study on stakeholders’ views on the participation of pregnant women in the APOSTEL VI study: a low-risk obstetrical RCT
Source: BMC Pregnancy Childbirth. 2019 Feb 11;19:65. doi: 10.1186/s12884-019-2209-7 (PMC6371564; doi:10.1186/s12884-019-2209-7)
Supplement: Supplementary file 1 — Box S1 a description of the APOSTEL VI study. (DOCX 22 kb) [file 12884_2019_2209_MOESM1_ESM.docx]

**Box S1.**

**APOSTEL VI**

The APOSTEL studies are a series of studies in the field of treatment of preterm labour within the Dutch Consortium for Healthcare Evaluation and Research in Obstetrics and Gynecology (NVOG Consortium 2.0.). The APOSTEL VI study in particular assesses whether a cervical pessary prolongs pregnancy in women who have been admitted for threatened preterm birth but remained undelivered after 48 hours (<http://www.studies-obsgyn.nl/apostel6>). Women are randomly allocated to receive either a cervical pessary or no intervention. Women participating in the study were not perceived to be at an increased risk since previous studies using the pessary had shown no foetal adverse effects and the cervical pessary was not associated with increased neonatal or maternal morbidity and mortality (APOSTEL VI Research Protocol). The REC of the UMC Utrecht classified the APOSTEL VI study as a low-risk study.

The APOSTEL VI study took place from November 2013 until September 2016, when the study was prematurely stopped following the advice of the Data and Safety Monitoring Board (DSMB). The premature cancellation was due to the fact that after interim analysis the intervention was unlikely to improve outcome, and maternal side effects were often present in the intervention arm.

Our qualitative study took place from March 2015 till September 2016. We interviewed pregnant women shortly after they were randomized to receive either the pessary or no intervention. We reached saturation before the APOSTEL VI itself was cancelled and in all our interviews it was therefore assumed that the APOSTEL VI would be completed. Following the premature cancellation of the APOSTEL VI study, we re-contacted the interviewed women and we asked them to reflect on their experiences in order to develop a comprehensive view of the research process.
