# Supplementary material for: A qualitative study on stakeholders’ views on the participation of pregnant women in the APOSTEL VI study: a low-risk obstetrical RCT
Source: BMC Pregnancy Childbirth. 2019 Feb 11;19:65. doi: 10.1186/s12884-019-2209-7 (PMC6371564; doi:10.1186/s12884-019-2209-7)
Supplement: Supplementary file 2 — Table S1-A: Topic list healthcare professionals. (DOCX 23 kb) [file 12884_2019_2209_MOESM2_ESM.docx]

**Topic list healthcare professionals^[[1]](#footnote-1)^**

What are the views and experiences of healthcare professionals regarding participation of pregnant women in clinical research?

**Research experience**

APOSTEL VI

- What is the aim of the study?
- What are the potential risks and benefits for the mother and the foetus?
- What is your assessment of these risks (high/low) in comparison with the potential benefits?
- What are your views on reaching the sample size?
- What is your role in the study?
- Can you describe the recruitment and consent procedure and your role in the process?
- Can you recall times and reasons for not informing a pregnant woman who was eligible for participation about the study?
- In your opinion, what are the motivations of pregnant women to participate or decline?

In general

- Can you tell me about your role and the types of research you are involved in?
- What are the differences and similarities between recruitment and consent procedures of different studies?
- Can you recall times and reasons for not informing a pregnant woman who was eligible for participation about a study?
- What are your considerations when assessing or designing a research protocol?
- Do you recall times and reasons why a research protocol received a negative assessment?
- How do you weigh potential benefits against potential risks?
- Do you recall decisions where the interest of the pregnant woman had priority over the interest of the foetus, or the other way around?
- How do research participants view risks of participating in your experience?
- What is your role in relation to participants’ views on risks?

**Participation of pregnant women in general**

There are different opinions regarding research participation of pregnant women. Some argue that we should not include pregnant women at all, while others argue that we should include pregnant women more often. Some even argue for a type of routine inclusion. Although there is no accepted definition of this term, it could mean a default of inclusion in research, unless there are scientific or ethical reasons for exclusion.

- What is your position in the debate regarding (routine) inclusion of pregnant women?
- What is your opinion about a) observational research, b) interventional research, c) drug trials (off-label/new medication), d) obstetric versus non-obstetric research involving pregnant women?
- What is your experience and view on pregnancy registries?
- What are your thoughts on including pregnant women in the different phases or research?
- What are your thoughts on including pregnant women during different phases of pregnancy?
- What is your opinion on prioritising research in pregnant women?

Risk

- Do you have suggestions on how to balance risks and benefits?
- What do you view as a minimal risk or a minor increase over minimal risk?
- In some trials there is a risk threshold that is called “minimal risk”; this means that the risks in the trial are comparable to risks in daily life or in standard clinical care (e.g. blood draws). What is your opinion about this “minimal risk” threshold for research in pregnant women?
- What is your opinion about trials where the risks are more than the minimal risk standard?
- Is there a maximum of acceptable risk for a pregnant woman or foetus in research?
- How do research risks that are allowed in research with children play a role?
- There is a difference between research where the research participant may benefit from participating, and research where the research participant has no benefit but it may be beneficial for future patients. Do you think the level of acceptable risks should differ between these types of research?

Vulnerability

Vulnerability is a term that is sometimes used in clinical research in relation to groups or persons who are at an increased risk of being harmed, for example because they are less able to protect their own interests. Some argue that pregnant women are vulnerable in research in comparison with other research participants and that they need special protection because of their vulnerability.

- What is your opinion on pregnant women’s vulnerability in clinical research?
- What are your suggestions regarding potential special protection?
- What is your opinion on pregnant women’s vulnerability outside of clinical research?

1. We performed this qualitative study as part of a larger study. The same research population and topic list was therefore used to answer two different research questions: stakeholder’s views on inclusion of pregnant women in the APOSTEL VI (this paper) and stakeholders’ views on acceptable levels of risk (reported elsewhere). [↑](#footnote-ref-1)
