# Supplementary material for: A qualitative study on stakeholders’ views on the participation of pregnant women in the APOSTEL VI study: a low-risk obstetrical RCT
Source: BMC Pregnancy Childbirth. 2019 Feb 11;19:65. doi: 10.1186/s12884-019-2209-7 (PMC6371564; doi:10.1186/s12884-019-2209-7)
Supplement: Supplementary file 3 — Table S1-B: Topic list pregnant women. (DOCX 24 kb) [file 12884_2019_2209_MOESM3_ESM.docx]

**Topic list pregnant women^[[1]](#footnote-1)^**

What are the views of pregnant women regarding their participation in clinical research?

**Participation in APOSTEL VI**

Recruitment

- You were recently asked to participate in this trial; can you go to the moment the trial was first mentioned to you?
- Can you explain how you experienced the course of the following process?
- Can you tell me the aim of the study?
- What motivations played a role in your decision to participate/decline?
- What motivation was ultimately decisive?
- How decisive was your decision?
- Who were involved in your decision? How important was their opinion?

Risks

- What are your reasons to say that this trial poses no or any risks?
- What are your views regarding the potential risks?
- I imagine that you made a certain calculation concerning the potential benefits and risks of participating in this trial. How did you do this?
- Can you hypothesise about your decision about this trial if there would not have been potential benefit for your baby, but it would only have been potentially beneficial for future patients?

**Participation of pregnant women in general**

There are different opinions regarding research participation of pregnant women. Some argue that we should not include pregnant women at all, while others argue that we should include pregnant women more often. Some even argue for a type of routine inclusion. Although there is no accepted definition of this term, it could mean a default of inclusion in research, unless there are scientific or ethical reasons for exclusion.

- What is your opinion regarding the inclusion and the routine inclusion of pregnant women?
- On what grounds would you consider participation in a trial?
- What is your opinion about a) observational research, b) interventional research, c) drug trials, d) obstetric versus non-obstetric research involving pregnant women?
- What are your reasons to say there is or there isn’t a difference between a drug trial and the trial you are currently enrolled in/you were asked to participate in?
- Have you previously been asked to participate in clinical research or other types of scientific research?
- What were your reasons to participate/decline at that time?

Risks

- What is your opinion about clinical research in pregnant women that poses potential risk for the mother and/or the foetus?
- Do you experience a difference between research that poses only risks for you and none for your baby; and research that poses risks for the both of you?
- In some trials there is a risk threshold that is called “minimal risk”; this means that the risks in the trial are comparable to risks in daily life or in standard clinical care (e.g. blood draws). What is your opinion about this “minimal risk” threshold for research in pregnant women?
- What is your opinion about trials where the risks are more than minimal? How much more?
- There is a difference between research where the research participant may benefit from participating, and research where the research participant has no benefit but it may be beneficial for future patients. Do you think the level of acceptable risks should differ between these types of research?

Vulnerability

Vulnerability is a term that is sometimes used in clinical research in relation to groups or persons who are at an increased risk of being harmed, for example because they are less able to protect their own interests. Some argue that pregnant women are vulnerable in research in comparison with other research participants and that they need special protection because of their vulnerability.

- What is your opinion on pregnant women’s vulnerability in clinical research?
- What is your opinion on pregnant women’s vulnerability outside of clinical research?

1. We performed this qualitative study as part of a larger study. The same research population and topic list was therefore used to answer two different research questions: stakeholder’s views on inclusion of pregnant women in the APOSTEL VI (this paper) and stakeholders’ views on acceptable levels of risk (reported elsewhere). [↑](#footnote-ref-1)
